# Assessment of the effect of phenytoin on cutaneous healing from excision of melanocytic nevi on the face and on the back

**DOI:** 10.1186/1471-5945-10-7

**Published:** 2010-08-24

**Authors:** Carlos Augusto Zanardini Pereira, Alice de Oliveira de A Alchorne

**Affiliations:** 1 Dermatologist of the Dermatology Service of Hospital Santa Casa de Curitiba, Pontifícia Universidade Católica do Paraná, Brazil; 2 Dermatologist, PhD in Dermatology, Post-graduation Course, Department of Dermatology, Universidade Federal de São Paulo, Brazil

## Abstract

**Background:**

Topical phenytoin is a powerful skin wounds healing and it may be useful in clinical practice. The purpose of this study was to evaluate the effect of topical phenytoin 0.5%, by comparing it with cream (control) in wounds resulting from excision of two melanocytic nevi in the same patient. Our purpose was also to assess if phenytoin had better therapeutic and cosmetic outcomes when compared with cream (control).

**Methods:**

This study evaluated 100 patients with skin wounds from excision of melanocytic nevi. 50 patients with lesions on the face and 50 patients with lesions on the back, totalizing 200 lesions excised with modified punch. The resulting superficial skin wounds had the same diameter and depth, and second intention healing followed.

Patients were followed for 60 days. Student's t-test, Mann Whitney nonparametric test, analysis of variance, LSD test, Shapiro-Wilks test and Fisher test were used to analyze the results, depending on the nature of the variables being studied.

**Results:**

Phenytoin showed better therapeutic and cosmetic results, by healing faster, with more intense epithelization in wounds in comparison with cream (control). Phenytoin showed a statistically significant difference regarding the following parameters (p < 0.05): wounded area and healing time. Phenytoin application resulted in a smaller area and a shorter healing time. Also the intensity of exudates, bleeding, and the epithelization were more intense in phenytoin-treated wounds. Regarding the shape and thickness of the scar, injuries treated with phenytoin had round and flat shaped scars in most of the cases. Considering patient's gender and phototype, female patients presented smaller wounds and scar areas; and phototype I had the largest scar areas. Contact eczema was an adverse reaction in 7 injuries located on the back caused by cream (control) and hypoallergenic tape.

**Conclusions:**

Phenytoin showed better therapeutic and cosmetic results compared with cream (control). Phenytoin is a low cost drug, which accelerates skin wounds healing in human patients. Trial registration: ISRCTN96539803

## Background

This paper investigates the use of topical phenytoin in the treatment of cutaneous wounds resulting from excision of melanocytic nevi on the face and on the back. The 5.5-diphenyl-2-4-imidazolidione, sodium (phenytoin), was synthesized in 1908, and has been used as an anticonvulsant, since 1937. It was later noticed that half the patients treated with phenytoin developed gingival overgrowth [1]. Local use of phenytoin speeds the healing process of cutaneous wounds. There are no studies using topical phenytoin (Table 1) in surgical wounds of human patients who returned for evaluation in 7, 14, 21 days (cosmetic outcome in the mediate postsurgical 60 days). Despite advances in skin healing treatments and the outbreak of new therapies, the treatment of trophic ulcers and wounds originated from the resection of skin tumors, remains a great challenge for doctors and patients. New therapeutic agents that improve the tissue repair process are needed, reducing healing time and preventing the formation of cheloids and retractile scar tissue. Those should also be cost effective and well tolerated for topic local use. Topical phenytoin has low systemic absorption [2].

**Table 1:** Preparation of a cream containing phenytoin

War N Lanett	20%
Volatile Silicone	2%

Grape seed oil	8%

Sorbitol	10%

Preservatives: (metilparabeno, propilparabeno, cosmoguard)	q.s

Phenytoin powder	0.5%

Distilled water	q.s to 100 g

## Methods

This blind, non-randomized, prospective, longitudinal, comparative study was performed in 100 patients, by resection of two lesions clinically compatible with melanocytic nevi measuring 0.4 to 0.6 cm in diameter. There were 50 patients with lesions on the face and 50 patients with lesions on the back, totalizing 200 lesions. All the patients read and signed an informed consent approved by the Institution's Ethics Committee

**Figure 1:**
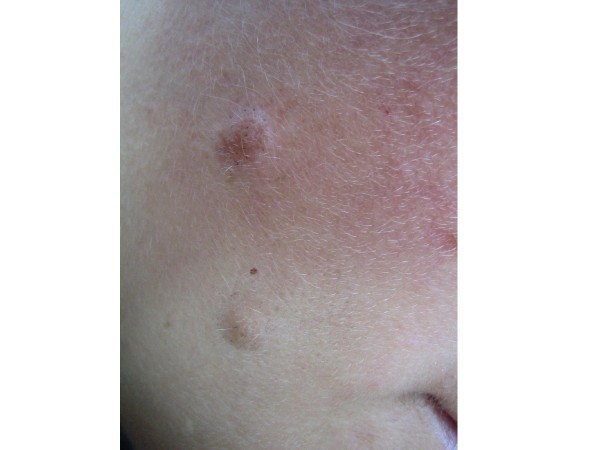
**Intradermal nevus**.

**Figure 2:**
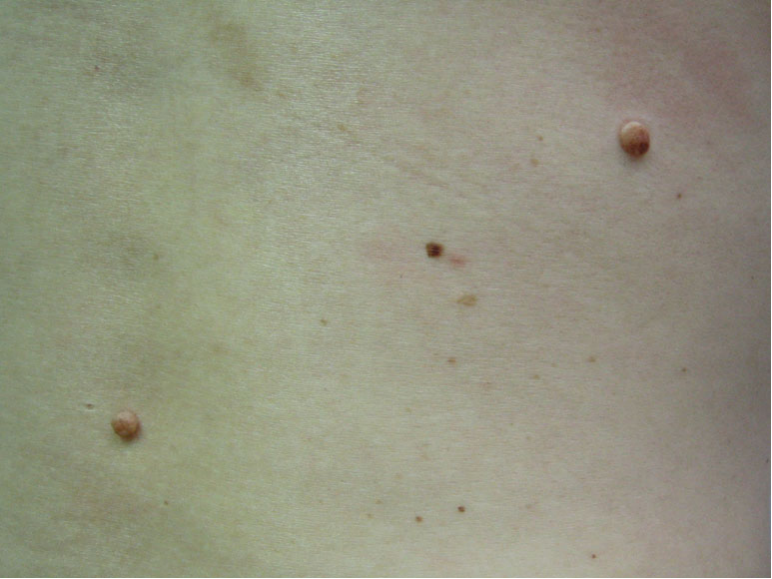
**Intradermal nevus**.

**Figure 3:**
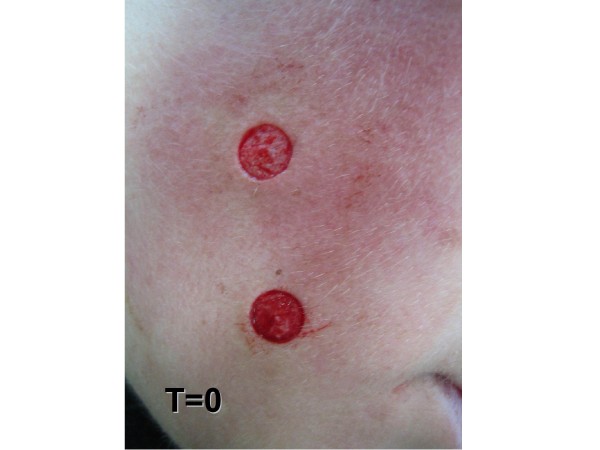
**Day of the surgical procedure (zero)**.

**Figure 4:**
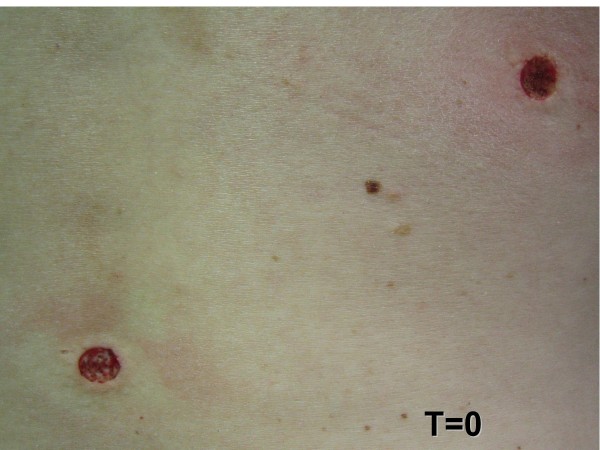
**Day of the surgical procedure (zero)**.

**Figure 5:**
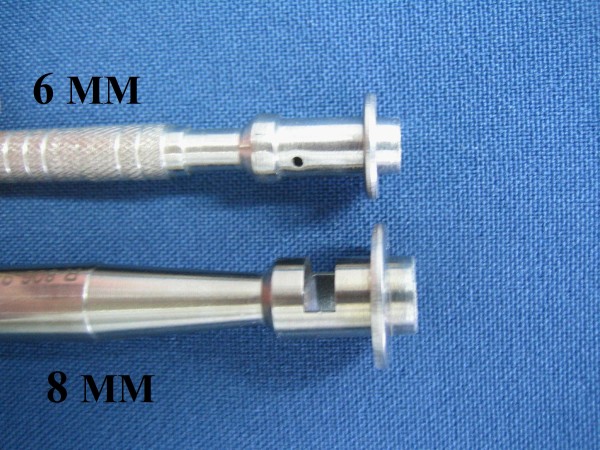
6 and 8 mm Punch.

Treatment methodology: inclusion criteria were healthy individuals of both sexes. This study included 29 male and 71 female patients, ranging from 16 to 77 years old, treated at *Hospital Santa Casa de Misericórdia*, in Curitiba. Inclusion criteria also comprehended patients being clinically healthy individuals of both sexes, with two melanocytic nevus lesions located in the face or back of the thorax, evaluated for possible surgical removal, when they presented at least one of the following changes: itching, changes in pigmentation, inflammation, bleeding, localization in an area of trauma, or aesthetic reasons. Wounds healing process, cosmetic outcome and possible complications were evaluated. All surgical procedures were carried out by the same dermatologic surgeon. Patients with immunosuppression, chronic renal insufficiency, and serious coagulopathies or with history of adverse effects caused by phenytoin were excluded. Data related to sex, age, skin type, and location of lesions were collected and are presented in Table 2. All patients returned for a clinical and cosmetic evaluation with photographic documentation in 7, 14, 21 and 60 days. All photographs were taken from a fixed distance of the wound. Only two melanocytic nevi of each patient were excised from the face or the back and received the same postsurgical treatment. Two nevi lesions with similar diameters, located on the face and on the back (figures 1 and 2) were excised with either 6 or 8 mm modified punches, depending on nevu's diameter, creating a superficial cutaneous wound, of same diameter and depth (figures 3 and 4). A modified punch (figure 5) was used for excising the lesions after local anesthesia with mepivacaine (10 mg/ml) + adrenaline (5 μg/ml). A 6 mm punch was used for lesions up to 0.4 cm and an 8 mm punch for lesions up to 0.6 cm. The resulting specimens were fixed in 10% formaldehyde for histopathologic examination. Bleeding was controlled mainly with compression. Hemostasis was achieved using low voltage electro-fulguration (2 W), in all cutaneous wounds. Following the surgical procedure, the wound was cleaned with water and soap. One of the injuries received a wound dressing with phenytoin and the other, a wound dressing with cream (control). All patients received two tubes of 20 grams of cream to make the dressing. One of the tubes containing only cream (control), marked in red and another tube with phenytoin 0.5%, marked in black. Patients received a sheet containing guidelines for wound dressing. Only the doctor knew which tube contained phenytoin. Patients were their own controls in this study. Full healing was defined as total closure of the injury without evidence of residual exudates or inflammation. The efficiency of the method was based on physician's evaluations and on comparative photographic documentation. A software was specially developed to automatically measure, in square millimeters, cutaneous wound and scars area, on digital images.

**Table 2:** Patients' details

	**No**.	%
Sex		

Female	71	71

Male	29	29

Total	100	100

Age (years) 16-77	100	100

Site of the lesion		

Face	50	50

Back	50	50

Skin type		

I	17	17

II	18	18

III	31	31

IV	12	12

V	2	2

* Skin type (Fitzpatrick's classification) [13]

### Statistical Analyses

Student's t-test was used to compare quantitative variables of two independent groups, and the Mann-Whitney nonparametric test was used for independent samples. To compare more than two groups, we used the analysis of variance with one factor and the LSD test, for multiple comparisons. For paired comparison of quantitative variables, the Student's t-test for dependent samples or the nonparametric test of Wilcoxon were used. The condition of variables for normality was evaluated by Shapiro-Wilks test. For dichotomous nominal variables, comparisons were made by use of the Fisher exact test or the binomial test. Values of p <0.05 were statistically significant.

## Results

**Figure 6:**
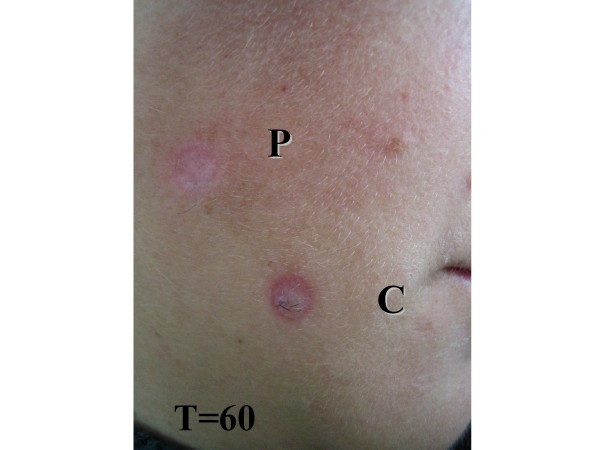
60 days after excision of nevi - P(phenytoin), C(cream control).

**Figure 7:**
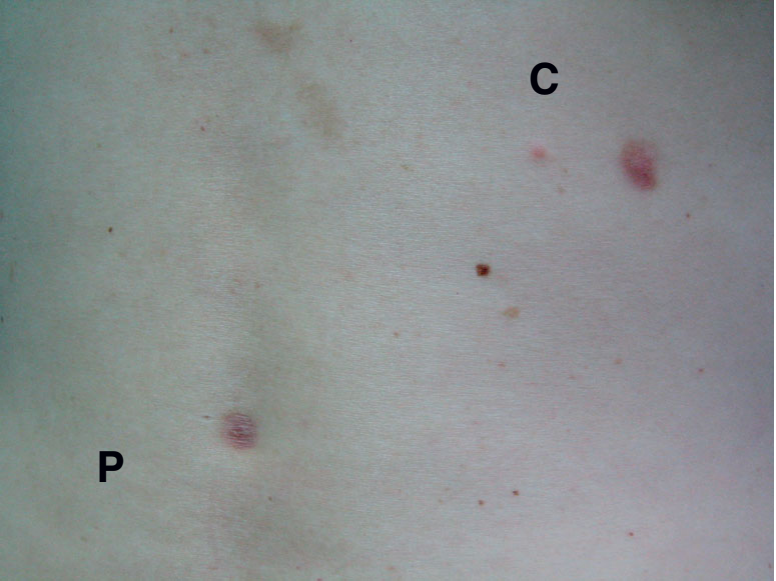
**60 days after excision of nevi - P (phenytoin), C (cream control)**.

Regarding epithelization, phenytoin treatment and cream (control) were statistically different (p < 0.05). Injuries treated with phenytoin showed more intense epithelization, with smaller wounded area and shorter healing time (Table 3 and 4). Considering the area and shape of the scar, there was a significant statistical difference in these parameters (p < 0.01). Most injuries treated with phenytoin were larger with flatter and round-shaped scars in comparison with injuries treated with cream. This happened because phenytoin inhibited skin retraction, leaving a larger scar, but a better cosmetic outcome. (Table 3 and figures 6 and 7).

**Table 3:** Results of the evaluation of 100 patients' wounds on the face and back Chronological data collection

	Cream	(control)				Phenytoin	Cream	
07^th^	14^th^	21^th^	60^th^	VARIABLES	07^th^	14^th^	21 th	60^th^
*16.97*	6.23	1.04	-------	Skin would area mm^2^	14.36	3.82	0.33	-------
------	------	------	13.95	Scar area mm^2^	------	------	------	14.82
D	F	G	H	Epithelization (intensity)	E	F	G	H
0	0	0	0	Hypertrophic tissue	0	0	0	0
+	+	+	0	Hyperemia	++	++	+	0
0	0	0	0	Infection	0	0	0	0
+	+	0	0	Bleeding (intensity)	++	++	0	0
------	-------	--------	A - C	Shape and thickness of the scar	--------	--------	--------	B
------	++	--------	-------	Adverse reactions. (allergic dermatitis)	-------	-------	--------	--------
+	+	0	0	Exudate (intensity)	++	+	0	0
--------	-------	---------	good	Cosmetic outcomes	--------	--------	----	excellent

This table was scored on a visual analogue scale: 0 (absent); + (mild); ++ (moderate); +++(severe)

Shape and thickness of the scar using the following scale: A = < 70% elliptic and flat; B = > 70% circular and flat; C = < 10% hypetrophic

For epithelization, the following scale was used: D = < 40% of the wound epithelized; E = > 60% epithelized wound with 6 cases of complete wounds epithelization; F = > 80% epithelized wound; G = total wound epithelization, thin epidermis; H = total wound epithelization, normal epidermis.

For cosmetic outcomes, the evaluation criteria was: excellent, no noticeable scar, the same color of the surrounding skin and no contractures should be visible; good, slightly noticeable scar with a different color from the surrounding skin and visible contractures; poor, noticeable scar, presence of depressed and/or intense dyschromia [10,14].

Adverse reactions (allergic contact dermatitis): ++ (7 cases on the back)

4 cases of contact dermatitis by cream (control) and 03 cases with the hypoallergenic tape

**Table 4:** Healing time and treatment performed

	Cream (control)		Phenytoin cream	
	
Healing time (days)	**No**.	%	**No**.	%
7	0	0	6	6
14	57	57	68	68
21	43	43	26	26
Total	100	100	100	100

Combining patient's gender with injured and scar areas, it was possible to establish a statistically significant difference for this parameter (p < 0.05). Female patients presented smaller wounded and scar areas (Table 5). Combining phototypes and scar area with matched comparisons of phototypes and scar area, and also matched comparisons of phototypes (I × II), (I × IV), (I × V), there was a statistical significance for this parameter (p < 0.05), showing the largest scar area for phototype I (Table 6). Considering presence of bleeding, hyperemia and exudates in wound, there was a statistically significant difference for these parameters (p < 0.05). Injuries treated with phenytoin cream had more bleeding, exudates and hyperemia, probably due to the vasodilatatory properties of the drug and neoangiogenesis. This study found that phenytoin has contributed to increase the number of capillaries and blood flow, also increasing exudates and redness, at the edges of the wound, in the initial phase of wound healing (Table 3). Just 6 out of the 100 patients had their wounds healed within a 7-day term. The injuries were on the face. All wounds received phenytoin application healing in 7 postoperative days, and the re-epitelization was faster in this group of patients. The patients were female; the average age was 47 years, three patients with skin type IV, one with skin type III, and two patients with skin type I. For excision of nevi three punches of 6 and 8 mm (Table 4 and 7) were utilized. Concerning the adverse reactions, allergic contact dermatitis occurred in 7 cases, located on the back (Table 8). The cosmetic outcome was considered excellent by the physician, for injuries treated with topical phenytoin and, for injuries treated with cream (control), it was considered good (Table 3 and figures 4 and 7).

**Table 5:** The association between patient's sex, skin wound area and scar area.

Variables	Sex	**No**.	Average	Minimum	Maximum	*P *value
Skin Wound area (7 days)	Female	71	12.99	4.12	30.25	
	Male	29	17.71	4.22	28.51	**0.006**

Skin wound area (14 days)	Female	71	3.36	0	11.94	
	Male	29	4.93	0.39	11.10	**0.039**

Skin wound area (21 days)	Female	71	0.29	0	3.97	
	Male	29	0.45	0	2.40	0.285

Scar area (60 days)	Female	71	13.88	4.77	23.17	
	Male	29	17.11	9.66	25.67	**0.004**

Student's *t*-test, for independent samples p < 0.05

**Table 6:** Skin type (Fitzpatrick's classification) [13]

Skin type under comparison	*P *value
I × II	0.296
I × III	**0.018**
I × (IV or V)	**0.012**
II × III	0.345
II × (IV or V)	0.165
III × (IV or V)	0.503

There are significant differences between skin type and scar area

**Table 7:** Patients with wounds healing in 7 days, using phenytoin

**No**.	Age (years)	Sex	Site of the lesion	Skin type	Punch (mm)
1	24	Female	Face	I	6
2	40	Female	Face	IV	8
3	41	Female	Face	IV	6
4	45	Female	Face	I	6
5	61	Female	Face	III	8
6	62	Female	Face	IV	8

*Skin type (Fitspatrick's classification) [13]

**Table 8:** Adverse reactions, causative agents of allergic contact dermatitis

Causative agents	**No**.	%
Cream (control)	4	57.2
Hypoallergenic tape	3	42.8
Total all adverse reactions	7	100.00

*adverse reactions on the back

## Discussion

In some studies the treatment of skin ulcers used phenytoin diluted in 0.9% NaCl, avoiding the formation of crust and burning that usually results from the direct application of phenytoin powder [2-4].

In a study with leprosy patients, and those with leg ulcers, one group was treated with topical phenytoin sodium suspension at a 2% concentration, and another group with topical phenytoin sodium suspension at a 4% concentration. Study result revealed no significant difference in the healing time of the ulcers [4].

Topical phenytoin can enhance wound healing in recalcitrant neuropathic diabetic foot ulcers of patients with no clinical evidence of ischaemia or infection [5].

In this study, the cream was used as a vehicle for the preparation of 0.5% topical phenytoin, and the vehicle cream was the control for the study, constantly hydrating the wound to avoid crusts or skin irritation. The author used topical phenytoin in the leg ulcers with excellent results. Excessive granulation tissue is often a result of long term phenytoin therapy, but it was not observed in our study [2,4,6]. All serum concentrations of phenytoin sodium dosed in patients that received topical phenytoin in these studies showed quite low or undetectable serum levels of the drug and the patients had no adverse effects during treatment [2,3,6]. The mechanisms by which phenytoin affects healing are poorly described. There are experimental studies showing that topical administration of phenytoin can promote wound healing by increasing collagen deposition, neovascularization, and the expression of growth factors in the wound tissue [7,8].

With aging, skin gradually loses its structure and functional characteristics, changing the wound healing process. Over time, important circulating hormones decline, steroids, and in particular androgens, play a predominant role [9]. In-vivo observations indicate that there is a sex divergence in the healing of acute skin wounds, and the healing process occurs more slowly in elderly male than in age-matched female [9].

In the event cosmetic results are not satisfactory for depressed scars or dyschromia, dermabrasion is an option to correct these complications. Another option is to perform a careful curettage, leveling the borders and the center of the scar. An intralesional infiltration with corticosteroids is efficient for hypertrophic scars [10].

Considering the effectiveness of phenytoin in accelerating wound healing, improving granulation tissue, reducing bacterial population at the ulcer surface area, and causing a fast and full healing, we recommend its use as a safe, effective, easy-to-use form, being also cost effective in the treatment of ulcers of several etiologies [11].

An experimental work concluded that phenytoin may be used locally to reduce scars, deformities and contractures in dystrophic epidermolysis bullosa [12]. In our study, topical phenytoin reduced, in most of the cases, the contracture of the surgical scar, with a round and flat shape. Nonetheless, we still need more comparative studies with other topical treatments to assess cutaneous wounds healing in human patients in order to confirm their benefits and clarify their mechanisms of action [1,12].

## Conclusions

Phenytoin showed a better therapeutic and cosmetic outcome in comparison with the cream (control). Phenytoin 0.5% cream is a low-cost drug with pharmacoeconomics advantages, which accelerates skin wounds healing with excellent cutaneous tolerability in human patients and cosmetic outcome. The dosage of topical phenytoin is an important variable that needs further investigation.

## Competing interests

The authors hereby state that there is no affiliation or significant financial involvement with any organization or entity interested in the subject or the materials discussed in the pages of this document.

## Authors' contributions

Each of the following served as study investigators: CP participated in the study design, data acquisition.

AA conceived of the study, participated in its design and coordination and helped to draft the manuscript. All authors read and approved the final manuscript.

## Pre-publication history

The pre-publication history for this paper can be accessed here:

http://www.biomedcentral.com/1471-5945/10/7/prepub
